# Expression of concern: Electrothermal patches driving the transdermal delivery of insulin

**DOI:** 10.1039/d5nh90012c

**Published:** 2025-02-20

**Authors:** Quentin Pagneux, Ran Ye, Li Chengnan, Alexandre Barras, Nathalie Hennuyer, Bart Staels, D. Caina, J. I. Avila Osses, Amar Abderrahmani, Valérie Plaisance, Valérie Pawlowski, Rabah Boukherroub, Sorin Melinte, Sabine Szunerits

**Affiliations:** a Univ. Lille, CNRS, Centrale Lille, Yncréa ISEN, Univ. Polytechnique Hauts-de-France, UMR 8520 – IEMN F-59000 Lille France sabine.szunerits@univ-lille.fr; b Univ. Lille, Inserm, CHU Lille, Institut Pasteur de Lille, U1011 – EGID F-59000 Lille France; c Institute of Information and Communication Technologies, Electronics and Applied Mathematics, Université catholique de Louvain 1348 Louvain-la-Neuve Belgium sorin.melinte@uclouvain.be

## Abstract

Expression of concern for ‘Electrothermal patches driving the transdermal delivery of insulin’ by Quentin Pagneux *et al.*, *Nanoscale Horiz.*, 2020, **5**, 663–670, https://doi.org/10.1039/C9NH00576E.

The Royal Society of Chemistry is publishing this expression of concern in order to alert readers that concerns have been raised regarding the reliability of the data. The Royal Society of Chemistry has asked the University of Lille to investigate this matter. An expression of concern will continue to be associated with the article until we receive conclusive evidence regarding the reliability of the reported data.

Heather Montgomery

7th February 2025

Managing Editor, *Nanoscale Horizons*

